# ABI1 regulates carbon/nitrogen-nutrient signal transduction independent of ABA biosynthesis and canonical ABA signalling pathways in *Arabidopsis*

**DOI:** 10.1093/jxb/erv086

**Published:** 2015-03-20

**Authors:** Yu Lu, Yuki Sasaki, Xingwen Li, Izumi C. Mori, Takakazu Matsuura, Takashi Hirayama, Takeo Sato, Junji Yamaguchi

**Affiliations:** ^1^Faculty of Science and Graduate School of Life Science, Hokkaido University, Kita-ku N10-W8, Sapporo 060-0810, Japan; ^2^Institute of Plant Science and Resources, Okayama University, Chuo 2-20-1, Kurashiki, 710-0046 Okayama, Japan

**Keywords:** Abscisic acid, C/N balance, FOX hunting system, nutrient signal, SnRK, sugar signal.

## Abstract

Plants are able to sense and mediate the balance between carbon (C) and nitrogen (N) nutrient availability to optimize metabolism and growth, described as the C/N response. To clarify the C/N signalling mechanism, C/N-insensitive plants were obtained from an *Arabidopsis* FOX hunting population, which over-expresses full-length cDNAs for individuals. The resulting *cni2-D* (*carbon/nitrogen insensitive 2-dominant*) plant was found to overcome the post-germination growth checkpoint and to expand green cotyledons in disrupted high C/low N stress conditions. The *CNI2* gene encodes ABI1, a phosphatase type 2C protein, which negatively regulates abscisic acid (ABA) signal transduction. Over-expressors of *ABI1* were found to be insensitive to disrupted C/N stress, whereas the loss-of function mutant *abi1-2* was hypersensitive, suggesting that ABI1 plays an essential role in the plant C/N response. By contrast, the C/N-dependent growth phenotype observed in wild-type plants was not associated with endogenous ABA content. Accordingly, the ABA-insensitive mutant *abi1-1*, which could not bind to the ABA–ABA receptor complex, was not insensitive and restored normal sensitivity to high C/low N stress. The canonical ABA signalling mutants *abi4* and *abi5* were also sensitive to disrupted C/N stress. Further gene expression analysis demonstrated that several genes in the SnRK2s and SnRK1s pathways are transcriptionally affected by high C/low N stress in wild-type plants regardless of the lack of increased endogenous ABA contents, whereas the expression of these genes were significantly suppressed in *ABI1 o*ver-expressors. Taken together, these results suggest direct cross-talk between C/N and non-canonical ABA signalling pathways, regulated by ABI1, in plants.

## Highlight

ABI1 was identified as the corresponding gene of the C/N-nutrient response mutant *cni2-D*. This study provides a new insight into the cross-talk between C/N and ABA signalling under the control of ABI1.

## Introduction

Plant growth and development are controlled by many environmental factors and stresses, including nutrition, light, drought, and osmotic stress. Carbon and nitrogen are essential for plants, being constituents of nutrients and metabolites that provide energy and serve as constitutive molecular backbones. Moreover, these constitutive molecules also possess hormone-like functions, transducing signals to regulate plant growth and development (Krouk *et al.*, 2010; Smeekens *et al.*, 2010; Stitt *et al.*, 2010). In addition to absolute amounts of cellular carbon (C) and nitrogen (N), the relative C/N balance, has been found critically to affect plant growth and development (Coruzzi and Zhou, 2001; Martin *et al.*, 2002; Sato *et al.*, 2009). Several genome-wide investigations have shown that carbon and nitrogen metabolites and signalling co-operatively control various pathways involved in plant growth and development, such as glycolysis/gluconeogenesis, the pentose-phosphate pathway, protein synthesis, protein degradation, protein targeting, and the regulation of protein activity (Palenchar *et al.*, 2004; Gutiérrez *et al.*, 2007). Despite the physiological importance of the C/N response, the molecular mechanisms mediated by C/N signals remain unclear.

To assess the molecular mechanisms mediating plant C/N responses, transgenic plants were screened for novel gain-of-function using the *Arabidopsis* FOX (*F*ull-length cDNA *O*ver-e*X*pressing) hunting system, which consists of independent transgenic lines expressing full-length cDNAs under the control of the CaMV promoter (Ichikawa *et al.*, 2006). A novel C/N-insensitive mutant plant was isolated called *carbon/nitrogen insensitive 1-D* (*cni1-D*), and it was found that the *CNI1* gene encoded the ubiquitin ligase ATL31 (Sato *et al.*, 2009). Over-expression of *ATL31* rescued plants from post-germination development arrest under extremely high C/low N stress conditions (Sato *et al.*, 2009). Subsequent analysis showed that ATL31 interacts with and ubiquitinates 14-3-3 and regulates plant growth via 14-3-3 degradation in response to C/N status (Sato *et al.*, 2011).

The phytohormone abscisic acid (ABA) is critical for plant growth in response to environment challenges such as drought, salt, and osmotic stress. Additional exogenous ABA delayed germination and arrested plant growth, similar to plants exposed to excess sugar stress (Dekkers *et al.*, 2008; Umezawa *et al.*, 2009). Genetic approaches have identified several sugar-insensitive mutants (Smeekens, 2000; Rook and Bevan, 2003), with many of these mutants found to be defective in ABA biosynthesis or ABA signalling, including *gin1*/*ABA2* (Laby *et al.*, 2000), *gin5*/*ABA3* (Rolland *et al.*, 2002), and *sun6*/*ABI4* (Huijser *et al.*, 2000). These findings showed close positive interactions between sugar and ABA signalling. Although the sugar–ABA response has been thoroughly investigated (Acevedo-Hernández *et al.*, 2005; Rolland *et al.*, 2006; Rook *et al.*, 2006), the relationship between C/N stress and ABA signalling has not yet been clarified.

To assess the molecular mechanisms involved in C/N signalling in higher plants further, C/N response mutants were screened and a novel FOX transgenic plant, *cni2-D* (*carbon/nitrogen insensitive 2-dominant*) was isolated which was able to survive under extremely high C/low N stress conditions. The *CNI2* gene encodes a type 2C protein phosphatase, ABI1, a negative regulator of ABA signalling. ABI1 is a central component of ABA signalling transduction, with its phosphatase activity inhibiting several SnRK2 proteins (Umezawa *et al.*, 2009; Vlad *et al.*, 2009). ABA and the ABA-receptor complex bind to ABI1 and inhibit its function when ABA is present (Park *et al.*, 2009), resulting in the activation of SnRK2s kinase activity and ABA signal transduction.

This study investigated the physiological function of ABI1 at the post-germination growth checkpoint in response to C/N, demonstrating that ABI1 negatively regulates C/N signalling. By contrast, quantification of ABA amounts and genetic analysis demonstrated that C/N signalling is not mediated by ABA biosynthesis and the canonical ABA signalling pathway that regulates sugar signalling through ABI4 and ABI5. These results provide new insight into the cross-talk between C and N signalling and its effect on the non-canonical ABA signalling pathway under the control of ABI1 protein.

## Materials and methods

### Plant materials and growth conditions

Wild-type *Arabidopsis thaliana* Columbia-0 (Col-0) and all other plant material used in this study were grown under the conditions described previously by Sato *et al.* (2009). The *Arabidopsis* FOX hunting population was provided by RIKEN (Ichikawa *et al.*, 2006). The ABA insensitive mutants *abi1-1* (Leung *et al.*, 1994; Meyer *et al.*, 1994), *abi1-2* (SALK_072009; Saez *et al.*, 2006), *abi4-102* (CS3837; Laby *et al.*, 2000), and *abi5-1* (line ID: CS8105; Finkelstein, 1994) were obtained from the Arabidopsis Biological Resource Center (Ohio State University, OH, USA). Surface-sterilized seeds were plated on modified MS medium. After stratification for 3 d at 4 °C in the dark, the plates were incubated at 22 °C with a 16/8h light/dark cycle.

### Isolation of the *cni2-D* plant

The *cni2-D* plant was isolated by screening *Arabidopsis* FOX lines with selection medium containing 300mM glucose and 0.1mM nitrogen as described previously (Sato *et al.*, 2009). The identity of the *ABI1* gene was determined by PCR using T-DNA primers that amplify the inserted cDNA fragment (Ichikawa *et al.*, 2006). The resulting PCR fragments were cloned into the pCR2.1 vector (Invitrogen, http://www.invitrogen.com) and sequenced.

### C/N response assay

Surface-sterilized seeds were sown on MS medium modified with different concentrations of glucose and total nitrogen, as described by Sato *et al.* (2009). The number of green-coloured cotyledons was counted 7 d after sowing. For transient limited-nitrogen treatment, seedlings were transferred to medium containing 0.3mM nitrogen after being grown for 7 d in control medium containing 3mM nitrogen.

### Plasmid constructions and plant transformation

A full-length *ABI1* cDNA fragment was amplified by PCR using the primers described in Supplementary Table S1 at *JXB* online. The fragment was sequenced and cloned into the pENTR/D-TOPO vector (Invitrogen) to generate the plasmid pENTR/ABI1. Full-length *ABI1* cDNA was subsequently introduced into the pMDC83 T-DNA binary vector (Curtis and Grossniklaus, 2003), according to the Gateway instruction manual (Invitrogen), placing the full-length *ABI1* gene under the control of the 35S promoter (*35S-ABI1*). This *35S-ABI1* construct was used to transform *Arabidopsis* as described by Sato *et al.* (2009).

### Transcript level analysis

Total RNA was isolated from plants as described by Sato *et al.* (2009), and 500ng RNA were reverse transcribed to cDNA with Super Script II (Invitrogen). RT-PCR analysis was performed with normalized cDNA samples for appropriate cycles, using the primer sets described in Supplementary Table S1 at *JXB* online. PCR products were electrophoresed on agarose gel and visualized by ethidium bromide staining. Quantitative RT-PCR (qRT-PCR) was performed using SYBR premix EX Taq (TAKARA) on an Mx3000P QPCR System (Agilent Technologies) according to the manufacturer’s protocol. The internal control for calculating ΔΔCt was *18S rRNA*. The specific primer sets used for qRT-PCR analysis are shown in Supplementary Table S2 at *JXB* online.

### Quantitative analysis of endogenous ABA content

The ABA contents of *Arabidopsis* plantlets were analysed essentially as described by Iehisa *et al.* (2014). Briefly, plantlets were grown for 7 d after germination in each C/N medium. Approximately 100mg of fresh weight of each were frozen in liquid nitrogen and ground into a fine powder by vigorously shaking with a vortex mixer in a 14ml round bottom plastic tube together with a 10mm Zirconia bead. ABA was extracted twice with 4ml of 80% (v/v) acetonitrile containing 1% (v/v) acetic acid and the internal standard (4ng d_6_-ABA_,_ Icon Isotopes, Summit, NJ, USA) at 4 °C for 1h. After clearing by centrifugation, the supernatant was evaporated and loaded onto an Oasis HLB column (Waters, Milford, MA, USA). The eluate containing ABA was evaporated and applied to an Oasis MCX column (Waters) to remove cationic compounds. After washing the column with 1% acetic acid, ABA was eluted with 80% acetonitrile containing 1% acetic acid. The eluate was evaporated and applied to an Osasis WAX column (Waters). After successive washes with 1% acetic acid and 80% acetonitrile, the acidic fraction containing ABA was eluted with 80% acetonitrile containing 1% acetic acid. This fraction was dried and dissolved in 1% acetic acid. ABA was determined by LC-MS/MS (Agilent 6410) using a ZORBAX Eclipse XDB-C18 column (Agilent).

## Results

### Isolation of a *cni2-D* transgenic plant able to tolerate C/N stress conditions

**Fig. 1.**
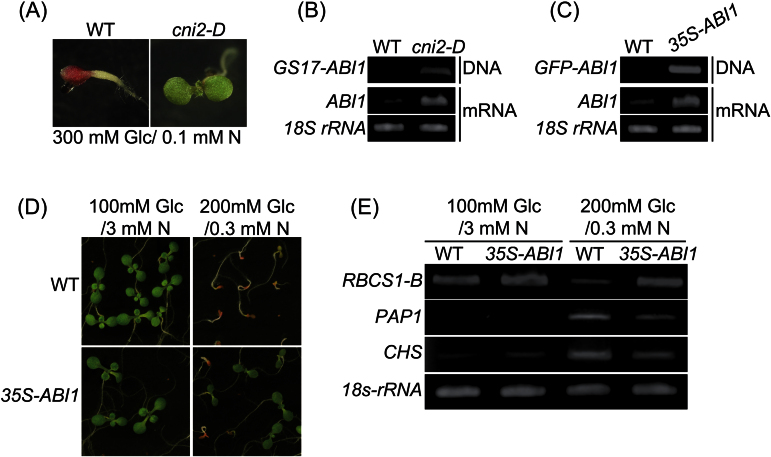
Isolation of *cni2-D* transgenic plant and C/N responses of *35S-ABI1*. (A) Screening of Arabidopsis FOX hunting population with medium containing 300mM glucose (Glc) and 0.1mM nitrogen (N). (B) Genomic PCR using primers for the *pBIG* vector (GS17) and *ABI1* (top panel) and RT-PCR of *ABI1* mRNA transcripts (middle panel) in WT and *cni2-D* plants. *18S rRNA* was used as an internal control (bottom panel). (C) Genomic PCR using primers for inserted *GFP* and *ABI1* (top panel) and RT-PCR of *ABI1* mRNA transcripts (middle panel) in WT and *35S-ABI1* plants. *18S rRNA* was used as an internal control (bottom panel). (D) Post-germination growth phenotypes of WT and *35S-ABI1* transgenic plants grown under normal (100mM Glc/3mM N) and high C/low N stress (200mM Glc/0.3mM N) conditions. Representative seedlings of four independent *35S-ABI1* lines are shown (see Supplementary Fig. S1 at *JXB* online). Images were taken 7 d after germination. (E) RT-PCR analysis of *RBCS1-B, CHS*,and *PAP1* mRNA transcripts in WT and *35S-ABI1* plants grown under the same conditions as in (D). *18S rRNA* was used as an internal control. WT, wild type (Col-0).

To assess the molecular mechanisms involved in the plant C/N response, FOX hunting populations were screened using medium containing an extremely high concentration of glucose (300mM Glc) and limited nitrogen (0.1mM N), termed high C/low N conditions. This screening resulted in the identification of a new C/N response mutant, *carbon/nitrogen insensitive 2-D* (*cni2-D*), which could continue post-germination growth in the extremely high C/low N medium (Fig. 1A). Under these conditions, wild-type (WT) plants showed severe growth defects and a strong purple accumulation of anthocyanin, whereas *cni2-D* plant could grow and generate green cotyledons. The full-length cDNA fragment inserted in the *cni2-D* FOX plant was recovered by genomic PCR using primers complementing the T-DNA construct. Sequencing of the recovered cDNA identified *At4g26080* as the *CNI2* gene, which encodes the protein ABI1, a Ser/Thr phosphatase type 2C (PP2C). This protein has been shown negatively to regulate ABA signalling by inhibiting downstream SnRK2s kinase activities (Raghavendra *et al.*, 2010; Umezawa *et al.*, 2010). Genomic PCR and RT-PCR analyses confirmed that the full-length cDNA of ABI1 had been inserted into, and was over-expressed in, *cni2-D* plants (Fig. 1B).

### Over-expression of the *ABI1* gene causes the C/N stress insensitivity seen in *cni2-D* plants

To confirm that the *ABI1* gene was responsible for the *cni2-D* phenotype, transgenic *Arabidopsis* plants over-expressing *ABI1* under the control of the CaMV 35S promoter (*35S-ABI1*) were grown in C/N stress medium. The transgenic nature of these plants was confirmed by genomic PCR and RT-PCR analyses (Fig. 1C). C/N response analysis was assessed in these plants grown under relatively mild high C/low N stress conditions (200mM Glc/0.3mM N), since the screening medium (300mM Glc/0.1mM N) was too severe for further analysis. Seeds of WT and *35S-ABI1* plants were sown in normal (100mM Glc/3mM N) and high C/low N stress (200mM Glc/0.3mM N) media. Although WT plants showed growth defects in this C/N stress medium, *35S-ABI1* plants expanded green cotyledons and continued post-germination growth (Fig. 1D; see Supplementary Fig. S1 at *JXB* online).

The transcript levels of marker genes responsible for the C/N response were analysed. In WT plants, the expression level of the photosynthesis-related *RBCS1-B* gene was lower in high C/low N stress conditions than in normal conditions, whereas expression of this gene in *35S-ABI1* plants was equal under both conditions (Fig. 1E). By contrast, the induction of the anthocyanin biosynthesis genes *PAP1* and *CHS* under high C/low N stress conditions was suppressed in *35S-ABI1* plants (Fig. 1E). These results demonstrate that over-expression of the *ABI1* gene causes the *cni2-D* phenotype, which is resistant to high C/low N stress.

### Over-expressor and loss-of-function mutant of the *ABI1* gene show reciprocal phenotypes under C/N stress condition

**Fig. 2.**
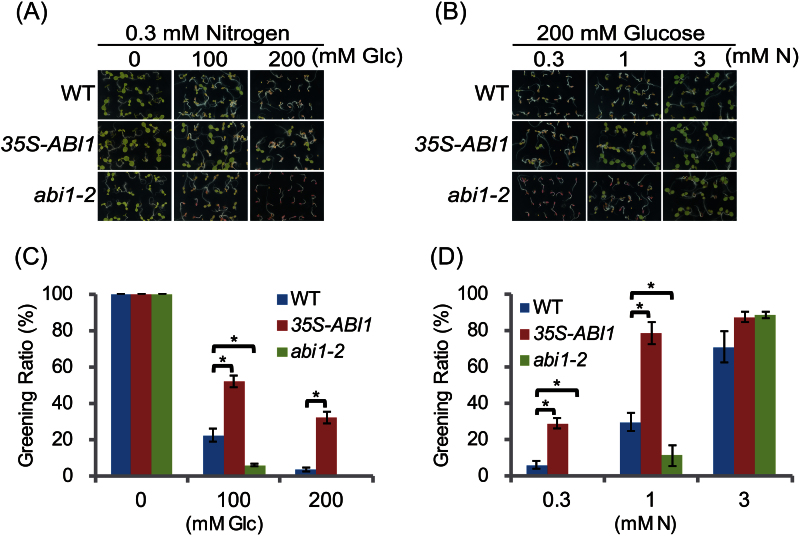
Post-germination growth of *35S-ABI1* and *abi1-2* plants under different C/N conditions. (A) Post-germination growth phenotype of WT, *35S-ABI1*, and *abi1-2* plants germinated on media containing 0.3mM N with 0, 100, and 200mM Glc. Images were taken 7 d after germination. (B) Post-germination growth phenotype of WT, *35S-ABI1*, and *abi1-2* plants germinated on medium containing 200mM Glc with 0.3, 1, and 3mM N. Images were taken 7 d after germination. (C) Greening ratios of WT, *35S-ABI1*, and *abi1-2* seedlings; growth conditions are described in (A). Each treatment involved 20–40 seedlings. Means ±SD of three independent experiments are shown. (D) Greening ratios of *35S-ABI1* and *abi1-2* seedlings grown under the conditions shown in (B). Each treatment involved 20–40 seedlings. Means ±SD of three independent experiments are shown. WT, wild type (Col-0). Asterisks indicate significant differences determined by Dunnet analysis (*P* <0.05).

To evaluate the function of ABI1 in the plant C/N response further, growth of the *35S-ABI1* and the loss-of-function mutant *abi1-2* was examined in parallel under several C/N stress conditions. Seeds of WT plants and *35S-ABI1* and *abi1-2* mutants were grown at constant nitrogen concentration (0.3mM) with various glucose concentrations (0, 100, and 200mM) and at constant glucose concentration (200mM) with various nitrogen concentrations (0.3, 1, and 3mM). Almost all WT, *35S-ABI1*, and *abi1-2* plants grew normally and expanded green-coloured cotyledons in 0mM Glc/0.3mM N medium (Fig. 2A). The post-germination growth of WT plants was inhibited and the number of individuals’ expanded green cotyledons was reduced to 22% in 100mM Glc/0.3mM N medium (Fig. 2A, C). The growth of WT plants was more severely inhibited and less than 5% of the WT plants showed any expansion of green cotyledons in 200mM Glc/0.3mM N medium. By contrast, the greening ratio of *35S-ABI1* plants was 52% in 100mM Glc/0.3mM N and 32% in 200mM Glc/0.3mM N (Fig. 2A, C), indicating that these plants were insensitive to increased glucose when nitrogen was limited. Growth inhibition was enhanced in the *abi1-2* mutants, with the greening ratio decreased to 6% in 100mM Glc/0.3mM N and 0% in 200mM Glc/0.3mM N (Fig. 2A, C). The growth inhibition due to increased Glc was not due to osmotic stress, because the WT and *abi1-2* plants did not show anthocyanin accumulation or growth defects in media containing 0.3mM N with 100 or 200mM mannitol (see Supplementary Fig. S2 at *JXB* online).

The effects of exogenous nitrogen availability on these mutants were examined in the presence of a constant amount of sugar. Although most WT, *35S-ABI1*, and *abi1-2* plants could grow normally and expanded green cotyledons in 200mM Glc/3mM N medium, WT plant growth was relatively inhibited and showed a decreased greening ratio (30%) in 200mM Glc/1mM N. This inhibition was more apparent in the *abi1-2* mutant (11%), while *35S-ABI1* plants were insensitive (78%) (Fig. 2B, D). Severe growth defects of WT and *abi1-2* mutants were observed in 200mM Glc/0.3mM N, while *35S-ABI1* was insensitive. Taken together, these results clearly demonstrate the reciprocal phenotypes of *35S-ABI* and *abi1-2* in response to increased Glc and limited N availability in the medium, suggesting that ABI1 plays an essential role in regulating plant growth in response to C/N status.

### ABA biosynthesis is not associated with the plant growth defect in response to C/N status

**Fig. 3.**
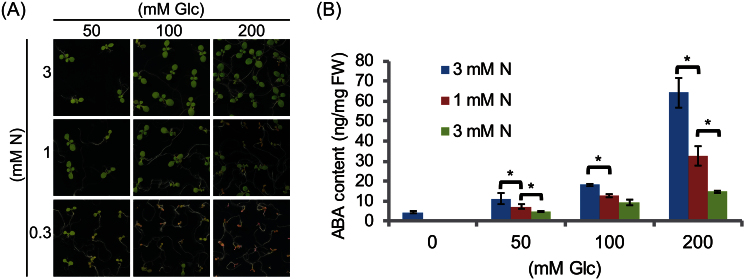
ABA content in response to C/N status. (A) Post-germination growth phenotype of WT plants grown in C/N medium containing combinations of 50, 100, and 200mM Glc and 3, 1, and 0.3mM N. (B) Endogenous level of ABA of WT plants grown under the C/N conditions indicated in (A) and 0mM Glc/3mM N. Seedlings were harvested 7 d after germination. Means ±SD of four independent experiments are shown. WT, wild type (Col-0). Asterisks indicate significant differences in response to limited N condition determined by Tukey analysis (*P* <0.05).

ABI1 is a phosphatase that is directly repressed by interaction with the ABA receptor PYR/RCAR in the presence of ABA, resulting in the activation of ABA signalling. The endogenous amounts of ABA have been reported to be increased by excess sugar in the medium, leading to growth arrest of *Arabidopsis* seedlings and indicating that endogenous sugar levels positively affect ABA biosynthesis. However, the relationship between C/N availability and ABA biosynthesis has not been determined. To determine whether the C/N-induced growth defect is caused by an increased ABA level, endogenous ABA contents were measured in WT *Arabidopsis* seedlings grown under each C/N condition. WT seedlings were grown in medium containing different concentrations of glucose (0, 100, 200, and 300mM) and nitrogen (0.3, 1, 3mM) for 7 d (Fig. 3A) and the ABA amounts were quantified by LC-MS analysis. ABA content was 5ng mg^–1^ FW in seedlings grown in 0mM Glc/3mM N and was significantly increased to 12, 20, and 63ng mg^–1^ FW during growth in medium containing 3 mM N with 50, 100, and 200mM glucose, respectively (Fig. 3B). Similar Glc-dependent increases in ABA content were observed at all N concentrations, although the effects were lower at lower N concentrations (Fig. 3B). Surprisingly, ABA contents were decreased in response to limited N. In the presence of 100mM glucose, ABA contents were estimated to be 20, 15, and 10ng mg^–1^ FW in seedlings grown in the presence of 3, 1, and 0.3mM N, respectively. Similar patterns were observed in other N-modified media containing 50mM and 200mM Glc. Interestingly, the ABA contents of seedlings grown in 100mM Glc/3mM N and 200mM Glc/0.3mM N were similar (Fig. 3B), despite their growth phenotypes being totally different (Fig. 3A).

**Fig. 4.**
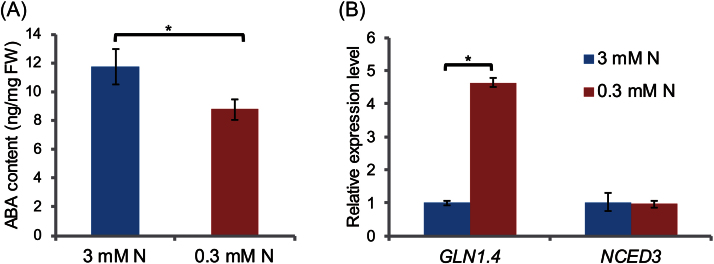
ABA amounts and *NCED3* expression in response to transiently limited N treatment. WT plants grown on control medium (100mM Glc/3mM N) for 7 d after germination were transferred to control N (3mM N) or limited N (0.3mM N) medium. Plants were harvested 3 d after transfer. (A) ABA quantification and (B) qRT-PCR analysis. Error bars represent SE (*n*=4). The level of expression of each gene was normalized relative to that of *18S rRNA* in the same sample and relative expression levels were compared with those of WT plants transferred to control N condition. Means ±SD of four independent experiments are shown. WT, wild type (Col-0). Asterisks indicate significant differences in response to limited N condition determined by Student’s *t* test (*P* <0.05).

To confirm that nitrogen limitation directly affects ABA biosynthesis, WT plants grown in control medium (100mM Glc/3mM N) for 7 d were transferred to N-deficient medium (100mM Glc/0.3mM N). After 3 d, the ABA content of plants grown in N-deficient medium was slightly but significantly lower than that of plants in control conditions (Fig. 4A), similar to the results observed at stable low N (Fig. 3B). Transfer to N-deficient medium also increased the expression of *Gln1.4* mRNA, which encodes a cytosolic glutamine synthase, a typical marker induced by N starvation, confirming that the endogenous N level of plants was limited (Fig. 4B). By contrast, the level of *NCED3* mRNA, which encodes a key enzyme for ABA biosynthesis, was not altered after the transfer (Fig. 4B). These results indicate that the growth defect observed in high C/low N stress medium is not due to an increased ABA content, suggesting that more complex regulatory mechanisms are involved under ABI1 control to mediate C/N conditions.

### ABA-insensitive mutants, *abi1-1*, *abi4*, and *abi5*, are not insensitive to C/N stress conditions

**Fig. 5.**
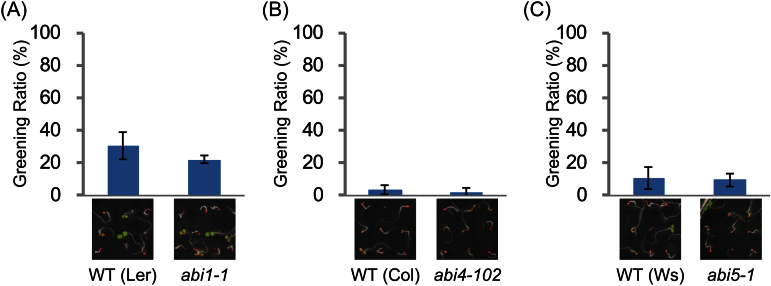
Post-germination growth of ABA-insensitive mutants under C/N stress condition. Greening ratio and post-germination growth phenotype of the ABA-insensitive mutants (A) *abi1-1* (B) *abi4-102*, and (C) *abi5-1* grown in high C/low N (200mM Glc/0.3mM N) conditions. WT plants for each mutant were Ler for *abi1-1*, Col-0 for *abi4-102*, and Ws-2 for *abi5-1*. Each treatment involved 20–40 seedlings. Means ±SD of three independent experiments are shown. Images were taken 7 d after germination.

Since the C/N stress response phenotype did not associate with the endogenous ABA content, the C/N response in the *abi1-1* mutant was examined. In *abi1-1*, the Gly180 residue is replaced by Asp, with the mutated ABI1 protein unable to bind to the ABA-receptor complex, resulting in constitutive inactivation of SnRK2 proteins (Santiago *et al.*, 2012). Thus, the *abi1-1* mutant was not insensitive to C/N stress and exhibited growth inhibition with anthocyanin accumulation, similar to WT plants (Fig. 5A). This result also suggests that the C/N response mediated by ABI1 is independent of ABA biosynthesis and is probably regulated by an alternative signalling cascade unlike typical ABA signalling cascades. To evaluate the C/N-responsive signalling pathway associated with ABI1, the C/N responses of *abi4* (*abi4-102*) and *abi5* (*abi5-1*) loss-of-function mutants were tested, since these genes encode key canonical transcriptional factors under the control of ABI1-SnRK2s (Raghavendra *et al.*, 2010). It had previously been shown that these *abi4* and *abi5* mutants are not resistant to the extremely high C/low N stress condition (300mM G/0.1mM N) used in *cni* mutant screening (Sato *et al.*, 2009). The detailed C/N responses of these mutant to relatively milder high C/low N stress conditions (200mM Glc/0.3mM N) was therefore re-examined and the ratio of green-coloured cotyledons was quantified. Both *abi4* and *abi5* mutants were sensitive to C/N stress, with both having similar greening ratios to WT plants (Fig. 5B, C).

These results strongly suggested that ABI1 controls plant C/N responses, but that these responses are mediated through a non-canonical typical ABA signalling pathway such as ABI4 and/or ABI5.

### Expression of ABA-related marker genes is differentially affected in response to C/N and is controlled by ABI1 regulation

**Fig. 6.**
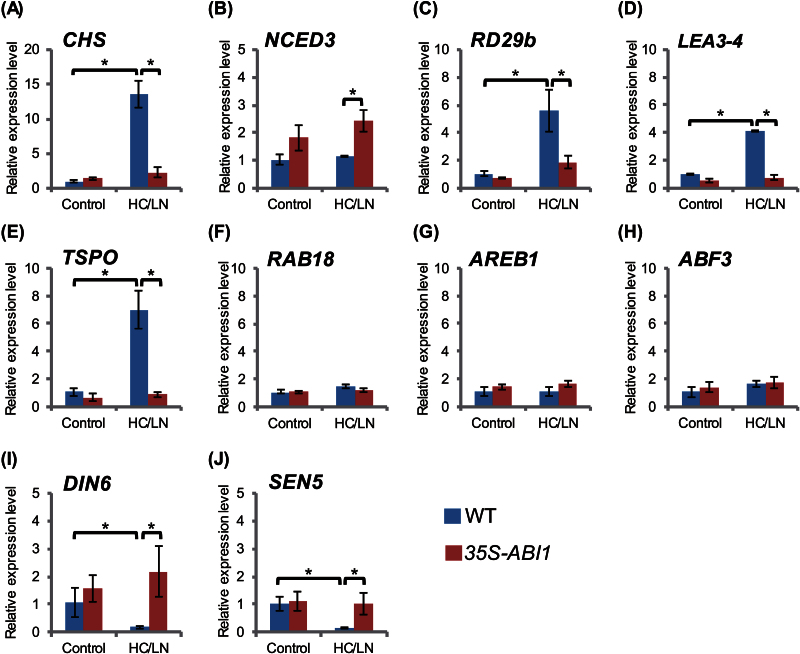
Transcripts levels of C/N- and ABA-related marker genes. WT and *35S-ABI1* plants were grown in normal (100mM Glc/3mM N; Control) or high C/low N (200mM Glc/0.3mM N; HC/LN) for 7 d after germination, and expression levels were analysed by qRT–PCR. The level of expression of each gene was normalized relative to that of *18S rRNA* in the same sample, and relative expression levels were compared with those of WT in normal C/N conditions. Means ±SD of three independent experiments are shown. WT, wild type (Col-0). Asterisks indicate significant differences determined by Tukey analysis (*P* <0.05).

To assess the C/N-mediated signal transduction pathway under ABI1 control, transcript analysis was performed in WT and *ABI1* over-expressor (*35S-ABI1*) plants. The expression of *CHS* was about 13-fold higher in WT plants grown under high C/low N stress (200mM Glc/0.3mM N) than under control (100mM Glc/3mM N) conditions, whereas *CHS* induction was clearly suppressed in *35S-ABI1* plants (Fig. 6A), confirming that ABI1 functions in C/N response. Moreover, the expression of *NCED3*, which encodes an ABA-biosynthetic enzyme, was not increased by high C/low N stress in WT plants (Fig. 6B), a finding consistent with the results of ABA quantification (Fig. 3B). The expression of *NCED3* was slightly higher in *35S-ABI1* than in WT plants, suggesting that ABA content is not responsible for the insensitive phenotype of *35S-ABI1* plants under the high C/low N stress condition. Evaluation of the expression of several ABA-responsive marker genes in the SnRK2s pathway (Mizoguchi *et al.*, 2010) showed that *RD29b* expression in WT plants was about 6-fold higher under high C/low N stress than under control conditions, but that *RD29b* expression was slightly lower in *35S-ABI1* plants under stress conditions (Fig. 6C). Similar expression patterns were observed for the *LEA3-4* and *TSPO* genes (Fig. 6D, E). By contrast, the levels of expression of *RAB18*, *AREB1*, and *ABF3* were not increased by high C/low N stress and were similar in WT and *35S-ABI1* plants (Fig. 6F–H). These results suggested that C/N stress activates some, but not all, of the ABA signalling cascade involving SnRK2s. SnRK1s-regulated marker genes, such as *DIN6* and *SEN5* (Baena-González *et al.*, 2007; Rodrigues *et al.*, 2013) were also evaluated since SnRK1s are important for energy homeostasis and recently reported to be direct targets of ABI1 (Rodrigues *et al.*, 2013). Transcript analyses showed that the levels of expressions of *DIN6* and *SEN5* in WT plants were significantly decreased in response to high C/low N stress, but were not affected in *35S-ABI1* plants (Fig. 6I, J). Taken together, these results suggest that high C/low N stress affects specific ABA-related signal transduction cascades under the control not only of SnRK2s but also of SnRK1s, and is independent of ABA biosynthesis.

## Discussion

### Identification of ABI1 as a C/N-response regulator

The *cni2-D* plants isolated in this study were insensitive to disrupted C/N-nutrient stress conditions, with *ABI1* being the gene responsible for the phenotype of these plants (Fig. 1). Over-expression of *ABI1* caused the successful post-germination growth of these plants under high C/low N stress condition, while the loss-of-function mutants showed hyper-sensitive responses and severe growth inhibition when compared with WT plants (Fig. 2), clearly demonstrating the importance that ABI1 plays in C/N signal regulation in *Arabidopsis*. *ABI1* encodes a protein phosphatase and functions as an essential negative regulator of ABA signal transduction. It has been reported that ABA is involved in multiple stress signal mediations including the sugar and osmotic signals during post-germination growth. Sugar and osmotic stresses enhance ABA biosynthesis followed by inhibition of ABI1 and then activation of the ABA signalling cascade (León and Sheen, 2003; Jossier *et al.*, 2009; Fujii *et al.*, 2011). Interestingly, however, ABA quantification analysis showed that endogenous ABA content is not correlated with growth inhibition in response to C/N status (Fig. 3). This finding was consistent with results showing that the *abi1-1* mutant, in which mutated ABI1 is unable to bind to the ABA-receptor complex, resulting in the constitutive inactivation of SnRK2s, was not resistant to C/N stress (Fig. 5). In addition, loss-of-function mutants of *ABI4* and *ABI5* were not insensitive to high C/low N stress conditions (Fig. 5), despite being resistant to high sugar stress and exogenous ABA at normal N concentration (Arenas-Huertero *et al.*, 2000). These results indicated that the high C/low N stress treatment used in this study is distinguishable from osmotic stress and that the C/N signalling cascade is not redundant to the canonical sugar- and ABA-signalling pathway under ABI1 control.

### ABA biosynthesis in response to N and C/N status

It was found that ABA biosynthesis is affected by C/N status but is not associated with plant growth phenotype in response to C/N (Fig. 3). ABA content was not increased under high C/low N stress conditions, although sugar promotes ABA biosynthesis. Both continuous and transient nitrogen limitation did not enhance the expression of *NCED3*, which encodes a key enzyme in ABA biosynthesis, under both high C/low N and limited-N conditions (Figs 4, 6).

Since little was known about the relationship between ABA biosynthesis and nitrogen availability, the results of this study are complicated. Related studies have reported that ABA biosynthesis in aerial parts of cucumbers is affected by limited nitrogen, but that this effect is dependent on time after treatment and developmental stage (Oka *et al.*, 2012). In *Arabidopsis*, transcription analysis showed that the expression of ABA biosynthetic genes could be activated by nitrogen starvation as well as by sugar supplementation (Yang *et al.*, 2011). By contrast, the ABA contents of shoots and roots were similar in *Arabidopsis* plants grown at high and low nitrogen conditions (Kiba *et al.*, 2011). Thus the effect of nitrogen on ABA biosynthesis may depend on developmental stage, N-treatment condition, and plant species, suggesting that a complex system regulates ABA biosynthesis in response to nitrogen availability. Although the direct participation of the ABA signalling pathway in nitrogen signal mediation remains unclear, genetic studies have demonstrated that the ABA pathway is involved in regulating plant development in response to nitrogen status. ABA-insensitive mutants such as *abi4* and *abi5* and ABA-deficient mutants were shown to be less sensitive to the inhibitory effects of high nitrate medium on lateral root formation (Signora *et al.*, 2001). Further studies are needed to determine the physiological function of the ABA pathway in mediating nitrogen availability and disrupted C/N stress.

### C/N signalling cascade under ABI1 control

The results in this study raise the question about how ABI1 regulates post-germination growth in response to C/N. To explore the unknown signalling pathway mediated by C/N response under ABI1 control, transcript levels of several ABA-signalling marker genes regulated by the SnRK2s pathway were examined. Interestingly, although endogenous ABA contents were not up-regulated, the levels of expression of *RD29b*, *LEA3-4*, and *TSPO* were increased by high C/low N stress, and suppressed in *ABI1* over-expressing plants (Fig. 6), suggesting a direct cross-talk between C/N and the ABA signalling pathway under ABI1 control. On the other hand, the levels of expressions of other marker genes, *RAB18*, *AREB1*, and *ABF3*, were not affected by high C/low N stress, indicating that C/N signals are mediated by specific ABA signalling pathways. In addition, the involvement of the SnRK1s, a family of essential kinases associated with vast transcriptional events and metabolic reprogramming, restoring homeostasis, and inducing tolerance to energy starvation stress (Baena-González and Sheen, 2008; Lunn *et al.*, 2014) was investigated. Recent studies showed that, besides SnRK2s, ABI1 could also directly target SnRK1s (Rodrigues *et al.*, 2013) and SnRK1s is involved in ABA signalling (Jossier *et al.*, 2009). Our transcript analyses demonstrated that high C/low N stress decreased the expression of *DIN6* and *SEN5*, both of which are typical SnRK1s-responsive marker genes, in WT plants, but which effect was clearly suppressed in the *ABI1* over-expressor (Fig. 6). This finding suggests the importance of the SnRK1s pathway in C/N signal mediation under ABI1 control. In responding to cellular energy status, SnRK1s target key components involved in protein synthesis and autophagic degradation (Smeekens *et al.*, 2010). Moreover, SnRK1s proteins have been reported to phosphorylate various 14-3-3 targeting proteins involved in primary carbon and nitrogen metabolism, including nitrate reductase (NR) and sucrose 6-phosphate synthase (SPS) (Comparot *et al.*, 2003; Smeekens *et al.*, 2010), suggesting a strong relationship to the C/N response. Our previous study revealed that 14-3-3 is targeted by ATL31 for ubiquitination and is essential for post-germination growth regulation in response to C/N (Sato *et al.*, 2011). Alternatively, a proteomics analysis showed that the SPS enzyme was precipitated along with ABI1 protein (Nishimura *et al.*, 2010), suggesting that ABI1 may regulate metabolic enzyme activity. Further biochemical and genetic analyses are required to understand the detailed functions of ABI1 and downstream SnRK proteins in the plant C/N response.

## Supplementary data

Supplementary data can be found at *JXB* online.

Supplementary Table S1. PCR Primers used for genotype and transcript analyses.

Supplementary Table S2. Primers used for quantitative RT-PCR analyses.

Supplementary Fig. S1. C/N response phenotype of *ABI1* over-expressors.

Supplementary Fig. S2. Osmotic stress response of *abi1-2* mutants.

Supplementary Data

## Abbreviations:

ABA: abscisic acid
C/N: carbon/nitrogen
cni: carbon/nitrogen-insensitive
FOX: full-length cDNA over-expressing
PP2C: protein phosphatase type 2C
SnRK: SNF1-related kinase.
