# ABI1 regulates carbon/nitrogen-nutrient signal transduction independent of ABA biosynthesis and canonical ABA signalling pathways in *Arabidopsis*

**DOI:** 10.1093/jxb/erv264

**Published:** 2015-06-04

**Authors:** Yu Lu, Yuki Sasaki, Xingwen Li, Izumi C. Mori, Takakazu Matsuura, Takashi Hirayama, Takeo Sato, Junji Yamaguchi

**Affiliations:** ^1^Faculty of Science and Graduate School of Life Science, Hokkaido University, Kita-ku N10-W8, Sapporo 060-0810, Japan; ^2^Institute of Plant Science and Resources, Okayama University, Chuo 2-20-1, Kurashiki, 710-0046 Okayama, Japan

*Journal of Experimental Botany*, Vol. 66, No. 9 pp. 2763–2771, 2015

doi:10.1093/jxb/erv086 Advance Access publication 20 March 2015

During the author correction cycle, one of the labels in Figure 3 was incorrectly changed from 0.3mM N to 3mM N. The corrected Figure 3 is reproduced below.



