# Supplementary material for: ABI1 regulates carbon/nitrogen-nutrient signal transduction independent of ABA biosynthesis and canonical ABA signalling pathways in Arabidopsis
Source: J Exp Bot. 2015 Mar 20;66(9):2763–71. doi: 10.1093/jxb/erv086 (PMC4986877; doi:10.1093/jxb/erv086)
Supplement: Supplementary Data [file supp_66_9_2763__index.html]

ABI1 regulates carbon/nitrogen-nutrient signal transduction independent of ABA biosynthesis and canonical ABA signalling pathways in Arabidopsis — ABI1 regulates carbon/nitrogen-nutrient signal transduction independent of ABA biosynthesis and canonical ABA signalling pathways in Arabidopsis — ABI1 regulates carbon/nitrogen-nutrient signal transduction independent of ABA biosynthesis and canonical ABA signalling pathways in Arabidopsis — Supplementary Data 

# ABI1 regulates carbon/nitrogen-nutrient signal transduction independent of ABA biosynthesis and canonical ABA signalling pathways in *Arabidopsis*

## Supplementary Data

Data files

**Files in this Data Supplement:**

- Supplementary Data - Supplementary Data
