# Supplementary material for: ABI1 regulates carbon/nitrogen-nutrient signal transduction independent of ABA biosynthesis and canonical ABA signalling pathways in Arabidopsis
Source: J Exp Bot. 2015 Mar 20;66(9):2763–71. doi: 10.1093/jxb/erv086 (PMC4986877; doi:10.1093/jxb/erv086)
Supplement: Supplementary Data [file supp_erv086_jexbot141218_file001.pdf]

Supplementary Table S1.
Primers used in PCR for genotype and transcript analyses.

	Forward Primer	Revers Primer
ABI1	GAATGGAGCTCGTGTTTTCGG	GTTCAAGGGTTTGCTCTTGAGT
18S rRNA	CGGCTACCACATCCAAGGAA	GCTGGAATTACCGCGGCT
RBCS1-B	ATGGCTTCCTCTATGCTCTCCT	TTAAGCATCAGTGAAGCTTGGG
CHS	CTTGACTCCCTCGTCGGTCA	CAAGACGACTGTCTCAACAGTG
PAP1	GCTCTGATGAAGTCGATCTTCT	CTACCTCTTGGCTTTCCTCTA
G17	GTACGTATTTTTACAACAATTACCAAC	
GFP		TCTTGAAGAAGTCGTGCCGCTT

Supplementary Table S2.
Primers used in quantitative RT-PCR analysis.

	Forward Primer	Revers Primer
18S rRNA	CGGCTACCACATCCAAGGAA	GCTGGAATTACCGCGGCT
CHS	AAGCGCATGTGCGACAAG	TCCTCCGTCAGATGCATGTG
Gln1;4	GATCTTTGAAGACCCTAGTGTTG	TTGGTTTAGGGTCTAGAGACAGA
NCED3	GCCGAAGATTCATCGAACAT	CGAGTTGATTCACCGGTTTT
RD29b	AAAAGAGAGGCACCGACTCA	CCGTTGACCACCGAGATAGT
RAB18	CGGATGTTTGTTTCTGGAGTG	CGGGGTTTTGTTTGAAGATG
LEA3-4	ACAAGACGGGTGGATTCTTGA	TGCATCAGCTGCACCCATT
TSPO	ACGCTCTTCGCTACGTATTTCC	ACGACGAGGACTTAGCTCGATT
AREB1	GCTTTGTCCAAGGTGCTTCTG	CAGGCGACACAGCACCAA
ABF3	GAATTCCGCAGAGGCAACA	CCAGCCCTGACCAAAAACTC
DIN6	TCCATCACTGCACGTCACTT	ATATTCCGCCACCTCTTTCC
SEN5	GCGAAACTCTCTCCGACTTC	CCACAGAACAACCTTTGACG

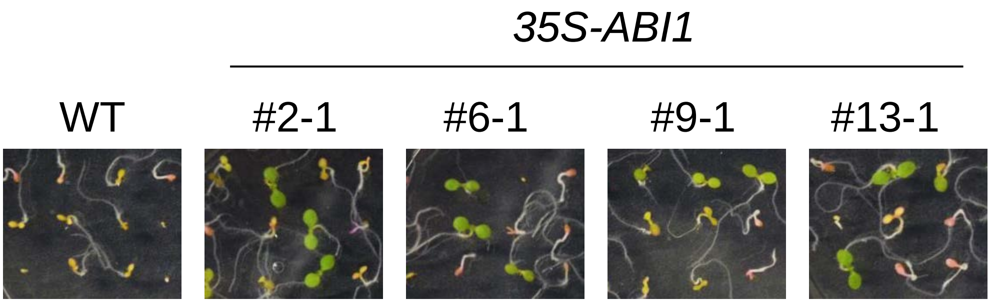

Fig. S1 C/N response phenotype of *ABI1* over-expressors.
Post-germinative growth of WT and four-independent lines of *35S-ABI1* plants grown on high C/low N stress medium (200 mM Glc/0.3 mM N). Images were taken at 7 days after germination. WT, wild-type (Col-0).

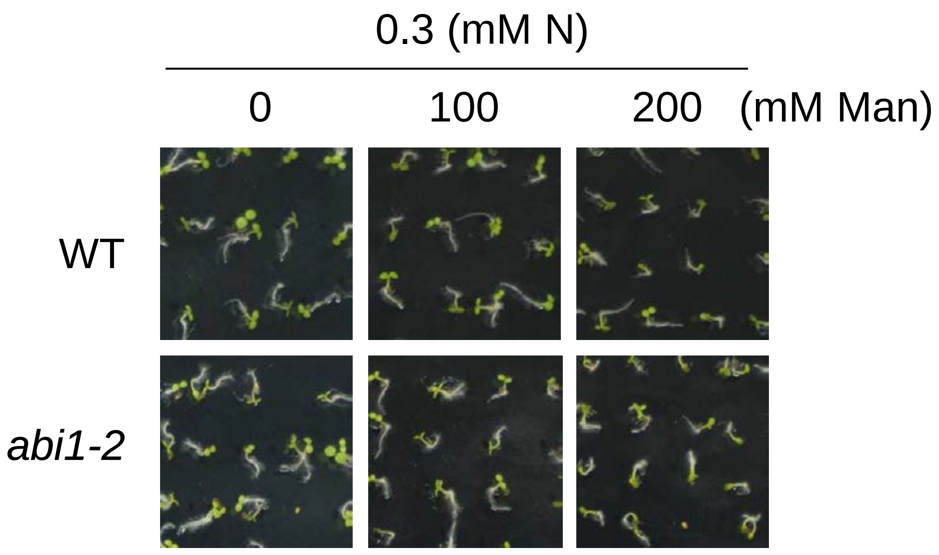

Fig. S2 Osmotic stress response of *abi1-2 mutants*

Post-germinative growth phenotypes of WT and *abi1-2* mutants grown on medium containing 200 mM mannitol (Man)/0.3 mM N. Images were taken at 7 days after germination. WT, wild-type (Col-0).
